# Plasma vesicle miRNAs for therapy response monitoring in Hodgkin lymphoma patients

**DOI:** 10.1172/jci.insight.89631

**Published:** 2016-11-17

**Authors:** Monique A.J. van Eijndhoven, Josée M. Zijlstra, Nils J. Groenewegen, Esther E.E. Drees, Stuart van Niele, S. Rubina Baglio, Danijela Koppers-Lalic, Hans van der Voorn, Sten F.W.M. Libregts, Marca H.M. Wauben, Renee X. de Menezes, Jan R.T. van Weering, Rienk Nieuwland, Lydia Visser, Anke van den Berg, Daphne de Jong, D. Michiel Pegtel

**Affiliations:** 1 Department of Pathology, Exosomes Research Group, and; 2 Department of Hematology, VU University Medical Center, Amsterdam, Netherlands.; 3 iZON Science, Oxford, United Kingdom.; 4 Department of Biochemistry and Cell Biology, Utrecht University, Utrecht, Netherlands.; 5 Department of Epidemiology and Biostatistics, VU University Medical Center, Amsterdam, Netherlands.; 6 Department of Functional Genomics, Center for Neurogenomics and Cognitive Research, Neuroscience Campus Amsterdam, VU University, Amsterdam, Netherlands.; 7 Department of Clinical Chemistry, Academic Medical Center, Amsterdam, Netherlands.; 8 Department of Pathology and Medical Biology, University of Groningen, University Medical Center Groningen, Groningen, Netherlands.; 9 Department of Pathology, Exosomes Research Group, VU University Medical Center, Amsterdam, Netherlands; ExBiome BV, Amsterdam, Netherlands.

## Abstract

**BACKGROUND.** Cell-free circulating nucleic acids, including 22-nt microRNAs (miRNAs), represent noninvasive biomarkers for treatment response monitoring of cancer patients. While the majority of plasma miRNA is bound to proteins, a smaller, less well-characterized pool is associated with extracellular vesicles (EVs). Here, we addressed whether EV-associated miRNAs reflect metabolic disease in classical Hodgkin lymphoma (cHL) patients.

**METHODS.** With standardized size-exclusion chromatography (SEC), we isolated EV-associated extracellular RNA (exRNA) fractions and protein-bound miRNA from plasma of cHL patients and healthy subjects. We performed a comprehensive small RNA sequencing analysis and validation by TaqMan qRT-PCR for candidate discovery. Fluorodeoxyglucose-PET (FDG-PET) status before treatment, directly after treatment, and during long-term follow-up was compared directly with EV miRNA levels.

**RESULTS.** The plasma EV miRNA repertoire was more extensive compared with protein-bound miRNA that was heavily dominated by a few abundant miRNA species and was less informative of disease status. Purified EV fractions of untreated cHL patients and tumor EVs had enriched levels of miR24-3p, miR127-3p, miR21-5p, miR155-5p, and let7a-5p compared with EV fractions from healthy subjects and disease controls. Serial monitoring of EV miRNA levels in patients before treatment, directly after treatment, and during long-term follow-up revealed robust, stable decreases in miRNA levels matching a complete metabolic response, as observed with FDG-PET. Importantly, EV miRNA levels rose again in relapse patients.

**CONCLUSION.** We conclude that cHL-related miRNA levels in circulating EVs reflect the presence of vital tumor tissue and are suitable for therapy response and relapse monitoring in individual cHL patients.

**FUNDING.** Cancer Center Amsterdam Foundation (CCA-2013), Dutch Cancer Society (KWF-5510), Technology Foundation STW (STW Perspectief CANCER-ID).

## Abstract 2

The extracellular RNA repertoire in circulating extracellular vesicles is useful indicator of therapy response and relapse in classical Hodgkin lymphoma patients.

## Introduction

Most, if not all, human tumors shed nucleic acids that end up in circulation, including DNA and mRNA/microRNAs (mRNA/miRNAs), collectively called extracellular RNA (exRNA) (1, 2). This realization has prompted intensive studies to demonstrate the biomarker potential of exRNA in cancer patients, as it holds notable advantages over classic tissue biopsy sampling. The current gold standard for cancer detection in blood is analysis of circulating tumor DNA (ctDNA) (2). The ctDNA approach is attractive, as it may directly reflect tumor tissue, for example by detection of actionable mutations (3) or rearranged genes (4). Because not all human tumor types in all stages carry the same mutations and ctDNA may not always reach high enough levels for robust detection in circulation, alternatives based on exRNA molecules are under development (5–7).

In the case of classical Hodgkin lymphoma (cHL), various limitations in analyzing ctDNA and/or exRNA can be considered. Most cHL tumors contain only a small proportion of malignant Hodgkin/Reed-Sternberg (HRS) cells of B cell origin, and the bulk of the tumor volume consists of nonmalignant cells, mostly reactive immune cells (8). The sensitivity for detection of tumor-specific translocations, mutations, and numerical chromosomal changes may therefore be less favorable in cHL than in some other malignancies. Because fragmented ctDNA is likely derived from apoptotic/necrotic tumor cells, with a half-life estimated between 2 and 12 hours, ctDNA may or may not signify vital tumor (9). This is a major benefit of fluorodeoxyglucose–PET/CT (FDG-PET/CT) imaging, which is currently used in clinical practice for cHL, as it detects active tumor and stroma. The reported positive predictive values of PET/CT in cHL patients are generally lower relative to the negative predictive values (10). TARC (thymus and activation-regulated chemokine, also known as cysteine-cysteine ligand 17 [CCL17]) is a chemokine produced by HRS cells that may have a role in shaping a favorable immune microenvironment (11, 12). TARC levels are increased in the sera of cHL patients and may also reflect tumor status, since elevated levels decrease during treatment in most clinical responders (13, 14).

Another class of cHL biomarkers are small noncoding miRNAs (15); miR155, in particular, is a strong candidate, as its precursor transcript *BIC* is amplified in the malignant HRS cells (16). miRNAs regulate genes that control differentiation and proliferation of human B cells, and perturbations in certain miRNA expression levels drive lymphomagenesis in vivo. For example, miR155 cooperates with c-MYC to prevent germinal center B cells from undergoing MYC-induced apoptosis (17). At least a small proportion of tumor-expressed miRNAs are actively secreted via extracellular vesicles (EVs) that are thus derived from living tumor cells (18, 19). EVs protect miRNAs from degradation by external RNAses and may help regulate gene expression in a non-cell-autonomous manner (20), promoting tumorigenesis (21). Moreover, EV-associated miRNAs may be more stable than other circulating miRNA fractions (22). For these reasons, EV-associated miRNAs may be enriched in defined lymphoma-associated miRNA species and could therefore overcome limitations of other approaches. Importantly, the advantages of EV-associated miRNAs as cancer biomarkers over measurements in total plasma or protein/HDL fractions have not been investigated.

Although cure rates for cHL patients are generally favorable, some patients will relapse or develop side effects from radiation/chemotherapy. Upcoming treatment strategies are aimed at personalized and targeted therapy that requires sensitive biomarkers suitable for repeated monitoring. In this study, we explored the optimal exRNA fraction for therapy response monitoring in a cohort of cHL patients by RNA sequencing (RNAseq) and qRT-PCR. The results suggest that EV-associated miR21-5p, miR127-3p, miR24-3p, let7a-5p, and miR155-5p report metabolic active tumor tissue with a high sensitivity. Analyzing serial samples from individual cHL patients before and after treatment and during long-term follow-up revealed that the levels of candidate miRNAs drop and stay low in clinical responders as determined with FDG-PET. The potential utility of an EV-based miRNA detection approach for response monitoring is discussed.

## Results

### Isolation of EVs from plasma using size-exclusion chromatography.

**Figure 1:**
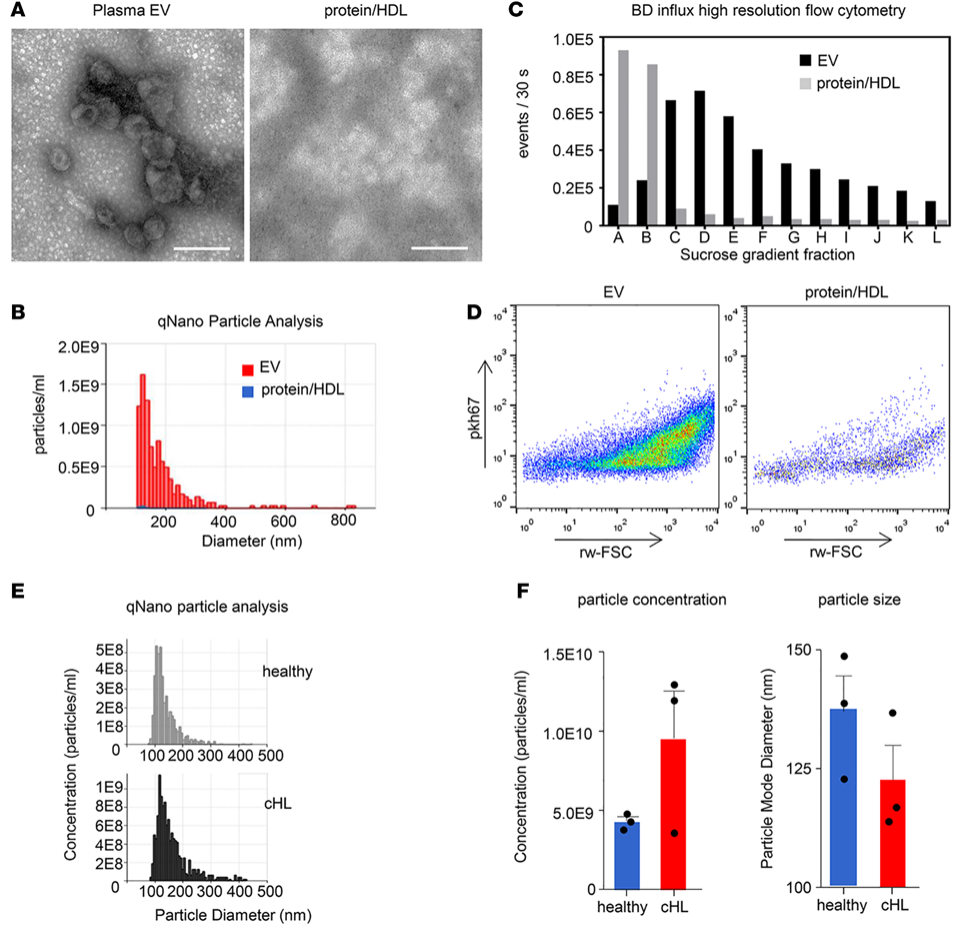
Size-exclusion chromatography separates extracellular plasma vesicles from protein/HDL. (**A**) EM images of plasma extracellular vesicles (EVs) and protein/HDL fraction after size-exclusion chromatography of 1.5 ml healthy donor plasma. Scale bar: 200 nm. (**B**) Particle analysis using qNano (iZON) of plasma EV (red) and protein/HDL fraction (blue). (**C**) High-resolution flow cytometry (BD Influx) of plasma EV (black) and protein/HDL fraction (gray) after pkh67 fluorescent labeling followed by sucrose gradient centrifugation. (**D**) Scatter plots of plasma EV and protein/HDL fraction corresponding with sucrose gradient fraction D, as shown in **C**. (**E**) qNano particle analysis of plasma EV from a healthy donor and a cHL patient. (**F**) Particle concentration and size using qNano. *n* = 3 healthy donors and cHL patients. Error bars represent mean ± SEM; dots indicate individual samples.

To measure putative lymphoma-associated miRNAs in circulation, we isolated EVs from plasma with size-exclusion chromatography (SEC) using a recently described procedure (23–25) (Supplemental Figure 1, A and B; supplemental material available online with this article; doi:10.1172/jci.insight.89631DS1). This method of isolation was chosen, as it may reduce the signal from abundant plasma miRNA associated with contaminating protein/HDL derived from nontumor-related sources; this method has been proven to outperform most other methods (23, 26, 27). Electron microscopy (EM) shows that vesicle-like membrane structures that range mostly from 50 to 200 nm are present in undiluted SEC vesicle fractions 9 and 10 but are absent in protein/HDL fractions 20 and 21 (Figure 1A). Particle analysis using qNano (iZON), based on tunable-resistive pulse sensing (TRPS), revealed that fractions 9 and 10 are enriched in particles with a size distribution that matches the EM images. Importantly, particles of this particular size range are close to absent in protein/HDL (Figure 1B). To further confirm separation of vesicle-like particles from other plasma components (protein/HDL, etc.) with another method, we performed high-resolution flow cytometry. Both vesicle and protein/HDL enriched SEC fractions (fractions 9 and 10, and 20 and 21, respectively) were labeled with PKH67, a fluorescent lipid dye that intercalates into lipid bilayers, and succumbed to sucrose gradient flotation to separate EVs from free dye and protein aggregates (28). In Figure 1C, we show that sucrose fractions C–E contain a highly enriched population of PKH67-positive particles (present in SEC fractions 9 and 10) when compared with protein/HDL (present in SEC fractions 20 and 21). Notably, the high levels of PKH67-positive events in protein/HDL enriched SEC fractions present in the high-density sucrose fractions A and B resemble nonfloating unincorporated PKH67 and protein aggregates (Figure 1, C and D) (28). We also performed comparative qNano particle analysis on a small panel of cHL patient plasma and plasma from healthy individuals. cHL patients seemed to have increased concentrations of circulating EVs, and the “additional” cHL-associated EVs are smaller in diameter (Figure 1, E and F). Together, these data show that EVs can be selectively isolated from healthy and cHL patient plasma using the SEC methodology.

### miRNAs are differentially distributed over plasma EVs and protein/HDL.

**Figure 2:**
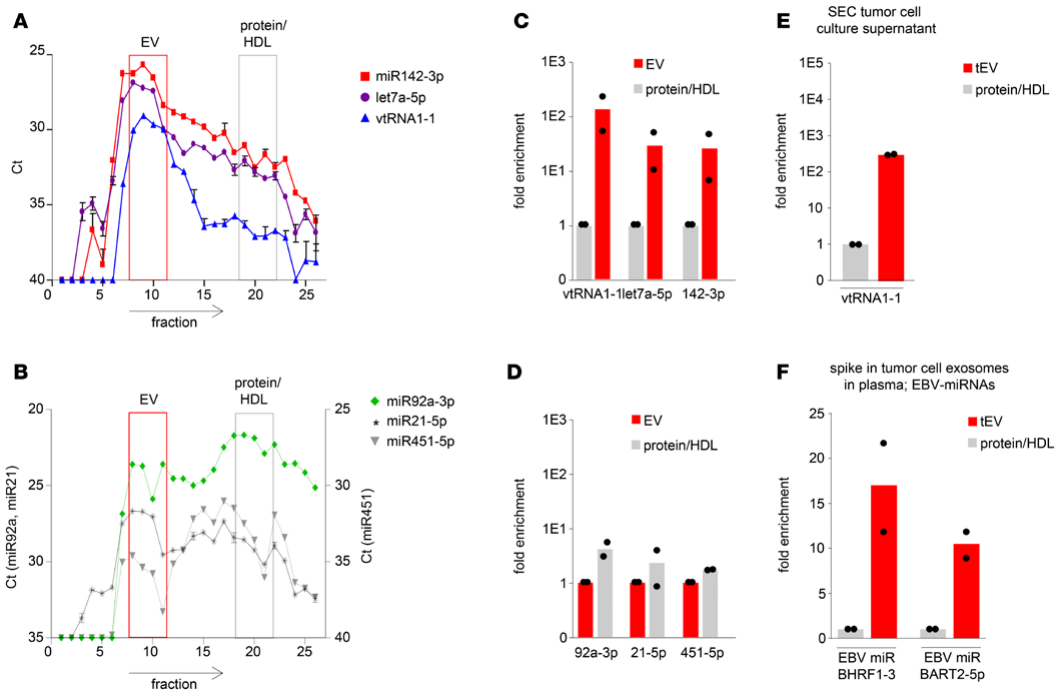
Small RNA distribution and recovery in EV fractions 9 and 10. (**A** and **B**) RNA distribution of miR142-3p, let7a-5p, and vtRNA1-1 (**A**) and miR92a-3p, miR21-5p, and miR451-5p (**B**) in 26 fractions upon size-exclusion chromatography (SEC) of 1.5 ml healthy donor plasma. Total RNA was isolated with TRIzol followed by RT-PCR. Data are depicted as raw Ct values; error bars represent SEM from PCR duplicates. (**C**) Fold enrichment of vtRNA1-1, let7a-5p, and miR142-3p in plasma extracellular vesicles (EVs) (fractions 9 and 10) compared with protein/HDL (fractions 20 and 21). Data are shown as the mean of 2 donors; dots indicate individual samples. (**D**) Fold enrichment of miR92a-3p, miR21-5p, and miR451-5p in protein/HDL (fractions 20 and 21) compared with plasma EVs (fractions 9 and 10). Data are shown as the mean of 2 donors; dots indicate individual samples. (**E**) Fold enrichment of vtRNA1-1 in tumor EV (tEV; fractions 9 and 10) compared with protein/HDL (fractions 20 and 21) after SEC of 1.5 ml B cell culture supernatant. (**F**) SEC of 1.5 ml healthy donor plasma after spike in with 50 μl tumor cell line–derived exosomes. Shown is the fold increase of EBV-miR BHRF1-3 and BART2-5p in EV (fractions 9 and 10) compared with protein/HDL (fractions 20 and 21). Data are shown as the mean of the 2 consecutive SEC fractions; dots represent individual fractions (**E** and **F**).

To determine the noncoding exRNA pool in plasma using stem-loop qRT-PCR, we compared the miRNA abundance in 26 fractions that were collected by SEC of 1.5 ml of healthy donor plasma. We measured the levels of the previously identified EV-associated miRNAs miR142-3p and let7a-5p (25) and the small ncRNA vtRNA1-1 previously found enriched in EVs (29). Both miRNAs and vtRNA1-1 were approximately 100-fold higher in vesicle-enriched SEC fractions 9 and 10 (Figure 2, A and C). In contrast, miR92a-3p, miR21-5p, and miR451-5p seemed more equally distributed over the different SEC fractions (Figure 2, B and D). When comparing plasma with sera, plasma appears better suited for separating vesicle-associated small exRNAs from the protein/HDL-bound exRNAs. Overall exRNA levels appear higher in plasma compared with sera when using an equal amount of volume as input material (Supplemental Figure 1C). To determine if the SEC method enriches for small RNAs, we analyzed 1.5 ml culture supernatant from transformed B cells for the vesicle-associated ncRNA vtRNA1-1 we found previously (29). As shown in Figure 2E, vtRNA1-1 is highly enriched in vesicle fractions 9 and 10 compared with protein/HDL fractions 20 and 21. To determine the recovery of tumor EVs in circulation using the SEC approach, we “spiked” purified lymphoma exosomes (from an Epstein-Barr virus–transformed [EBV-transformed] B cell line) in 1.5 ml plasma from a healthy EBV-negative donor. In Figure 2F, we showed that the recovery of exosome-associated EBV miRNAs BHRF1-3 and BART2-5p is 10- to 15-fold higher in EV fractions 9 and 10 compared with protein/HDL fractions 20 and 21. Thus, single-step SEC is a robust method to enrich for lymphoma cell–secreted EVs and useful for detection of associated miRNAs.

### RNAseq identifies lymphoma-associated miRNAs in plasma vesicles of cHL patients.

**Figure 3:**
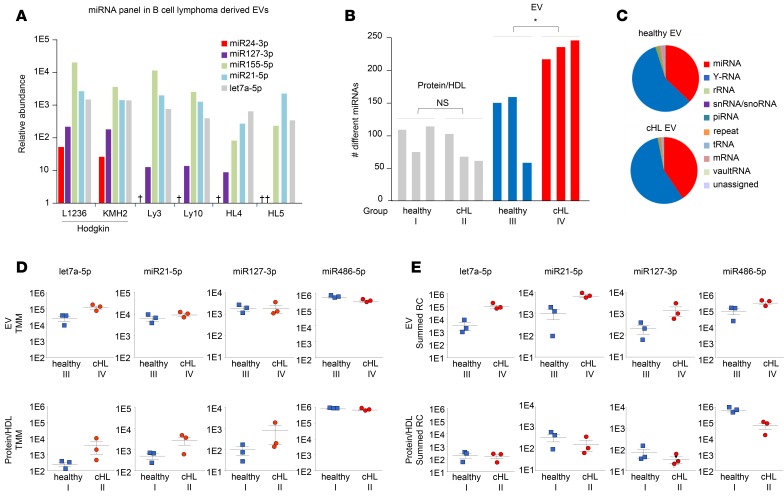
RNAseq reveals lymphoma-secreted miRNAs in circulating extracellular vesicles. (**A**) RNAseq analysis of miR24-3p, miR127-3p, miR155-5p, miR21-5p, and let7a-5p in extracellular vesicles (EVs) isolated from B cell lymphoma cell lines. Data are shown as reads per million miRNA reads (RPM). (**B**) Number of different miRNAs identified in plasma EV and protein/HDL fractions of healthy donors and cHL patients (*n* = 3 each). *P < 0.05 (unpaired 2-tailed *t* test). (**C**) Distribution of small RNA subclasses in plasma EVs of a healthy individual and a cHL patient. Data shown are of 1 representative donor (*n* = 3) and depicted as percentage read counts. (**D**) RNAseq analysis of let7a-5p, miR21-5p, miR127-3p, and miR486-5p in healthy and cHL patient plasma EVs and protein/HDL fractions. Dots represent individual samples; error bars represent mean ± SEM. Reads are normalized using the trimmed mean of M values (TMM) method. (**E**) As in **D**, but data are shown as summed read counts.

To identify EV-associated miRNAs in EV fractions, we performed small RNAseq on purified EVs from 6 different lymphoma cell lines that are of B cell origin. We discovered a high number of reads (reads per million) of miR155-5p, miR21-5p, and let7a-5p that are highly expressed in lymphoma tissue and involved in lymphomagenesis (5, 16, 30, 31). Moreover, we found that miR127-3p and miR24-3p are abundant in EVs from the Hodgkin lymphoma cell lines L1236 and KMH2 (Figure 3A). Next, we verified whether these candidate miRNAs could be identified in plasma EV fractions from cHL patients. Total RNA was isolated from cHL patients and healthy individuals and analyzed using an Agilent small RNA chip (Supplemental Figure 1D). The bioanalyzer profiles have a typical small RNA shape, as seen before in purified EVs (32). With a HiSeq 2500 (Illumina) device, 12 libraries were measured from 4 sample groups: (group I) protein fractions from 3 healthy subjects, (group II) protein fractions from 3 cHL patients, (group III) EV fractions from 3 healthy subjects, and (group IV) EV fractions from cHL patients. The libraries were sequenced (single-end 50-bp read length), and reads were mapped to the human genome with the sRNAbench small RNA analysis pipeline we developed previously (29). The analysis yielded a total of 15 million reads that were, however, unevenly distributed between the 4 groups (Supplemental Table 1) but similar between individual samples within each group. The distribution of small RNA classes was comparable in the EVs from cHL patients and healthy controls (Figure 3C), with 16%–67% of the total mapped reads belonging to the class of miRNAs in these groups (groups III and IV). In protein fractions (groups I and II), the large majority of read counts belonged to the class of miRNAs reaching 90% in the protein fractions of healthy subjects (Supplemental Figure 2A). We identified the most miRNA species (415 species) in the EV fractions from cHL patients (group IV) compared with EVs from healthy individuals (202 species) (group III). Importantly, the amount of different miRNAs identified in each cHL EV sample was significantly higher than that in healthy EV samples (*P* = 0.030), while, in each protein fraction, we detected fewer than 120 different species (Figure 3B). The numbers of different miRNAs detected in each library did not correlate with the total miRNA read counts, suggesting that the differences are not merely caused by sequence depth but may reflect pathological conditions (Supplemental Figure 2E). In all groups, miR486-5p and miR92a-3p accounted for the majority of miRNA reads (Supplemental Table 1), and these were the most pronounced in the protein fractions (Supplemental Figure 2B). This suggested that we detect more comprehensive miRNA repertoires of cHL patients in EVs compared with protein-bound miRNAs.

Next, we wished to verify whether RNAseq analysis could identify lymphoma-associated miRNAs in EVs and/or protein/HDL fractions. We first applied a differential expression method connected to EdgeR software that uses a trimmed mean of M values method for normalization. Consistent with the TaqMan RT-PCR data in Figure 2A, let7a-5p, an EV-associated miRNA, was highly enriched in EV groups III and IV, compared with the protein samples (groups I and II). Moreover, the normalized levels of let7a-5p are significantly higher in cHL-associated EVs (group IV) compared with control EVs (group III) (*P* = 0.03), while let7a-5p levels in protein fractions of patients (group II) and healthy controls (group I) were generally lower and not significantly different (*P* = 0.27) (Figure 3D). While miR21-5p in EVs of healthy individuals appears enriched compared with protein (confirming TaqMan PCR in Figure 2B), we only found a small difference in miR21-5p protein levels between patients and controls, while no differences are seen in either fraction for miR127-3p or miR486-5p (Figure 3D).

Because the optimal normalization method(s) for analysis of plasma RNAseq data is not established, we and others reasoned that an equal volume input allows for comparisons to identify disease-associated alterations in miRNA levels (5, 33). Interestingly, the lymphoma-associated miRNAs let7a-5p, miR21-5p, and miR127-3p have elevated reads, comparing EV samples from cHL patients and healthy controls, while miR486-5p reads are equally abundant (Figure 3E). Moreover the read counts of EV-related miR142-3p (25) are high in the EVs, corresponding with RT-PCR data. Although miR142-3p was barely detected in the protein fractions by RNAseq, RT-PCR revealed low but detectable amounts (Figure 2A and Supplemental Figure 2D). The summed reads representing cHL-associated miR24-3p and miR155-5p were elevated in patient EVs, while miR10b-5p values stood out as being particularly low in the cHL protein samples (Supplemental Figure 2D).

To determine if a correlation exists between the increased level of cHL-associated miRNAs and a subtype of plasma EVs, we performed RT-PCR analysis of 4 candidate biomarkers and determined concentration and size distribution with a qNano device (TRPS). As shown in Supplemental Figure 2C, the combined level of miRNAs (miR127-3p, miR155-5p, miR21-5p, let7a-5p) corresponds with EV concentration, while a reverse correlation with EV size is noticeable in cHL patient plasma. These correlations were much less pronounced in healthy EVs.

In summary, RNAseq reveals that read numbers of a small panel of lymphoma-related miRNAs are increased in EVs from cHL patients, which was not seen in protein fractions. These differences could be related to a different EV composition and/or content in cHL patient plasma compared with healthy individuals. While these conclusions require further validation, RNAseq-based miRNA discovery in patient plasma can be readily performed using SEC-purified EV fractions as an exRNA source.

### Candidate biomarker miRNAs are elevated in plasma vesicles of cHL patients compared with healthy individuals.

**Figure 4:**
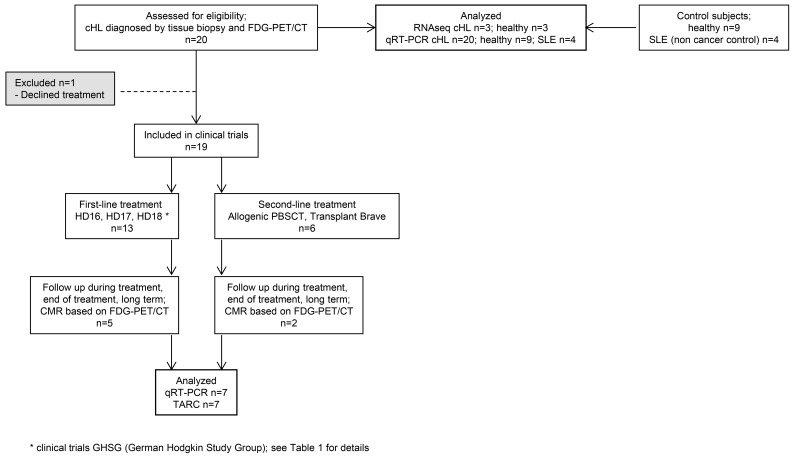
Study flow chart.

**Figure 5:**
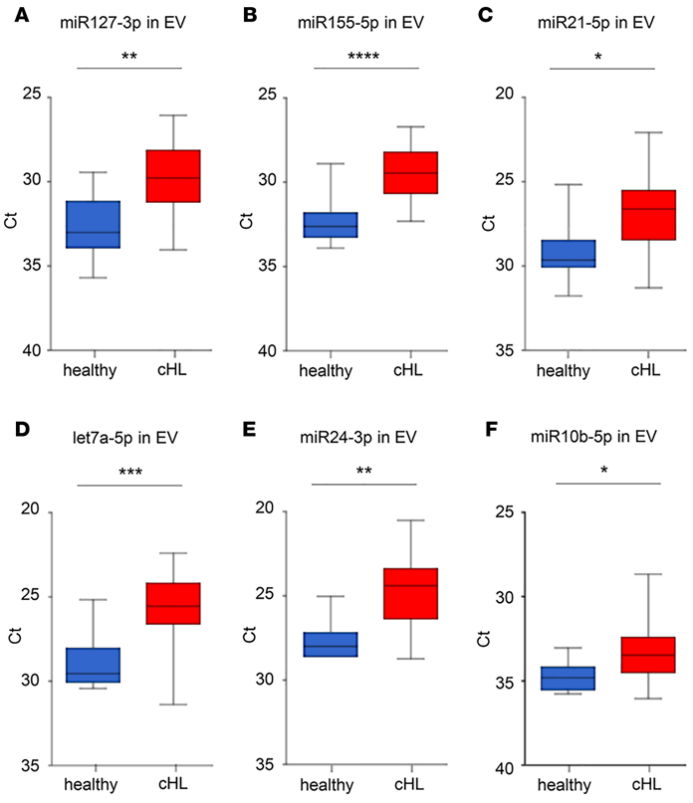
Candidate miRNA levels are elevated in EVs of cHL patients compared with healthy controls. RT-PCR analysis of miR127-3p (**A**), miR155-5p (**B**), miR21-5p (**C**), let7a-5p (**D**), miR24-3p (**E**), and miR10b-5p (**F**) in plasma extracellular vesicles (EVs) of healthy individuals (*n* = 9) and cHL patients (*n* = 20) after size-exclusion chromatography (SEC) and total RNA isolation using TRIzol. For each individual sample, the mean Ct value of SEC fractions 9 and 10 was used. Boxes show the 25%–75% percentile; whiskers show the minimum-maximum; and lines represent the median. *P < 0.05; **P < 0.005; ***P < 0.0005; ****P < 0.0001 (unpaired 2-tailed *t* test).

The RNAseq and RT-PCR data, combined with reports in the literature (5, 29, 30, 31, 34), suggested that miR127-3p, miR155-5p, miR21-5p, let7a-5p, and miR24-3p may represent noninvasive biomarkers for cHL. Next, we isolated EVs with the SEC method from 20 cHL patients prior to treatment and 9 healthy control subjects. The patient characteristics and requirements for inclusion in clinical trials are summarized in Table 1 and Figure 4. We extracted RNA from equal plasma volumes and determined miR127-3p, miR155-5p, miR21-5p, let7a-5p, and miR24-3p levels by the TaqMan (stem-loop) qRT-PCR method. We detected significantly higher levels of all candidate markers in patient samples compared with healthy controls (*P* = 0.0019, < 0.0001, 0.010, 0.0009, and 0.0017, respectively) (Figure 5, A–E). We also measured the level of the known oncomiR miR-10b that is not known to be associated with cHL. Although we observe a slight increase, this is much less pronounced than for the lymphoma-associated miRNAs (Figure 5F).

As an additional control, we measured the miRNA levels in SEC-isolated plasma EVs from patients with a systemic autoimmune disease but did not detect any difference with the healthy controls (*P* = 0.76 [miR127-3p], 0.73 [miR155-5p], 0.88 [miR21-5p], and 0.25 [let7a-5p] (Supplemental Figure 3, A–D). Moreover, we did not observe different levels for the miRNA panel in healthy female and male subjects (Supplemental Figure 3, E–H). Importantly, we also detected high levels of cHL-associated miRNAs in plasma EV fractions isolated from relapsed cHL patients (*n* = 7) compared with healthy controls (*P* = 0.013 [miR127-3p], 0.0022 [miR155-5p], 0.012 [miR21-5p], 0.0013 [let7a-5p], and 0.016 [miR24-3p], respectively) (Supplemental Figure 4, A–E). In contrast, the levels of the control oncomiR miR10b-5p (Supplemental Figure 4F) are more in range with those of healthy subjects, which is consistent with RNAseq data (Supplemental Figure 2D). These observations strongly suggest that the panel of cHL-associated miRNAs could be useful for monitoring treatment response and relapse. In fact, when we used miR-10b levels for normalization, the levels of miR127-3p, miR155-5p, miR21-5p, let7a-5p, and miR24-3p were significantly increased in the cHL patient population (*P* = 0.0025, 0.0005, 0.013, 0.0030, and 0.0050, respectively) (Supplemental Figure 4, G–K). Thus, the differences observed are not due to more or less input of EV and or RNA material, indicating our procedure of isolation and miRNA detection is robust.

**Figure 6:**
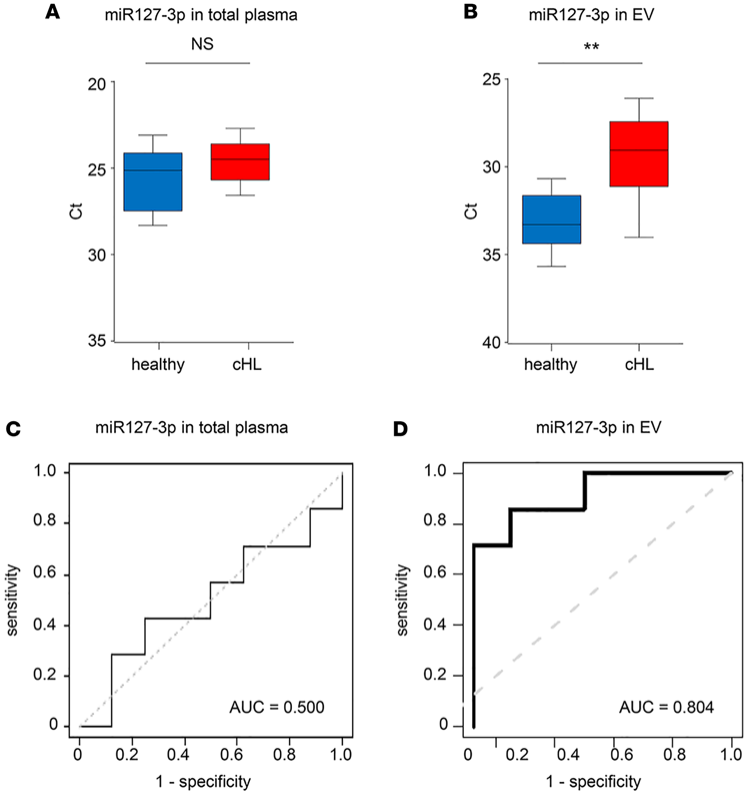
miR127-3p EV outperforms total plasma in distinguishing cHL patients from controls. (**A**) RT-PCR analysis of miR127-3p in total plasma of healthy controls (*n* = 7) and cHL patients (*n* = 8) after RNA isolation using TRIzol-LS. (**B**) RT-PCR analysis of miR127-3p in extracellular vesicle (EV) fractions of the same healthy individuals and cHL patients as in **A** after size-exclusion chromatography (SEC) and total RNA isolation. For each individual, the mean Ct value of SEC fractions 9 and 10 is used. (**A** and **B**) Boxes show the 25%–75% percentile; whiskers show the minimum-maximum; and lines represent the median. **P < 0.005 (unpaired 2-tailed *t* test). (**C** and **D**) ROC curves of miR127-3p in total plasma (**C**) and EV fractions (**D**) of the same healthy individuals and cHL patients as in **A** and **B**.

To ascertain whether it is important to measure the miRNA pool in plasma EVs specifically rather than in total plasma, we compared both approaches for miR127-3p and miR155-5p. In a side-by-side comparison of the same sample set we showed that total plasma miR127-3p levels are not significantly different between patients and controls (*P* = 0.21) (Figure 6A). In contrast, measurements in the EV fraction of the same patients showed that miR127-3p is significantly elevated (*P* = 0.0056) (Figure 6B). While miR155-5p was significantly increased in cHL patients versus controls when measured in total plasma and EV fractions (*P* = 0.0027 and 0.0030, respectively), fold differences were more robust in EVs (Supplemental Figure 5, A and B). Importantly, in a different patient cohort and using total sera as exRNA source, miR155-5p levels did not differ between cHL patients and healthy individuals (*P* = 0.16) (Supplemental Figure 5C). We determined the sensitivity and specificity of miR127-3p in distinguishing cHL patients before therapy from healthy individuals using ROC analysis. For miR127-3p, there seemed to be a substantial advantage in using plasma EV fractions as miRNA source (AUC = 0.80) (Figure 6D), since the same measurements in total plasma had no diagnostic value (AUC = 0.50) (Figure 6C). Thus, EV-associated circulating miRNAs seem more connected to the presence of Hodgkin’s disease than the protein-bound miRNA pool.

### miRNA levels in plasma vesicles of cHL patients correspond with clinical response to treatment.

**Figure 7:**
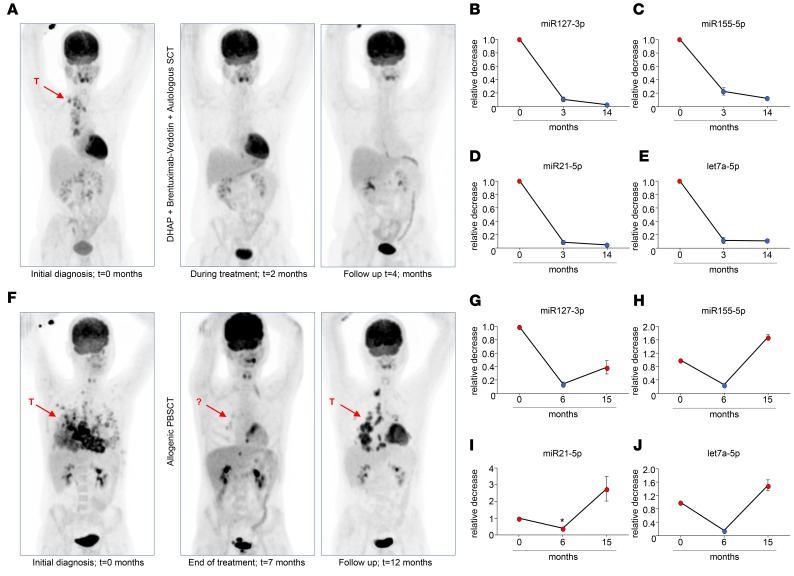
Candidate miRNA markers in plasma EVs correspond with clinical response to treatment. (**A**) FDG-PET imaging of a cHL patient at initial diagnosis, during treatment, and at follow-up 4 months after initial diagnosis. T, tumor. Arrows indicate the location of tumor masses at initial diagnosis. (**B**–**E**) RT-PCR analysis of miR127-3p (**B**), miR155-5p (**C**), miR21-5p (**D**), and let7a-5p (**E**) in plasma extracellular vesicles (EVs) of the same cHL patient as in **A** at diagnosis (*t* = 0), at the end of treatment (*t* = 3 months), and at long-term follow-up (*t* = 14 months). Data are shown as a relative decrease compared with *t* = 0. Dots represent the mean ± SEM of the 2 consecutive size-exclusion chromatography fractions. (**F–J**) As in **A–E**, but for a cHL patient who did not reach complete metabolic response. The asterisk indicates a relative low decrease compared with other patients measured.

FDG-PET is the standard method for detecting tumor mass and monitoring clinical response in cHL patients during active treatment and follow-up. The FDG-PET images depicted in Figure 7A show a reduction in vital tumor mass in a chemotherapy refractory patient. This patient was treated with second-line chemotherapy, DHAP, and the antibody-drug conjugate brentuximab-vedotin and subsequently received an autologous stem cell transplant. We measured substantial decreases in miR127-3p, miR155-5p, miR21-5p, and let7a-5p in the plasma EV fraction during and after treatment (*t* = 3 months) corresponding to FDG-PET status. Importantly, the miRNA levels remained stable for more than a year (*t* = 14 months) after initial diagnosis (*t* = 0). The decreased levels of these miRNAs measured in EVs during treatment are therefore unlikely related to secondary effects of the treatment (Figure 7, B–E). Similar results were observed in patients with primary tumor during and after first-line treatment (data not shown). In one patient that presented with relapsed disease, FDG-PET imaging revealed disappearance of metabolic tumor activity after allogenic stem cell transplant; however, after 12 months, tumor tissue reappeared, suggesting incomplete metabolic remission (Figure 7F). Notably, the levels of miR127-3p, miR155-5p, and let7a-5p in plasma vesicles initially decreased to “normal” levels upon treatment; miR21-5p stood out, as it dropped by 60%, while, on average, levels dropped >80%–90% in patients with complete metabolic response (CMR). In full correspondence with PET imaging, 3–4 miRNAs were increased dramatically at 15 months (Figure 7, G–J). These data illustrate that plasma vesicle-associated miRNAs are useful for monitoring cHL patients, as they reflect the presence of vital tumor tissue.

**Figure 8:**
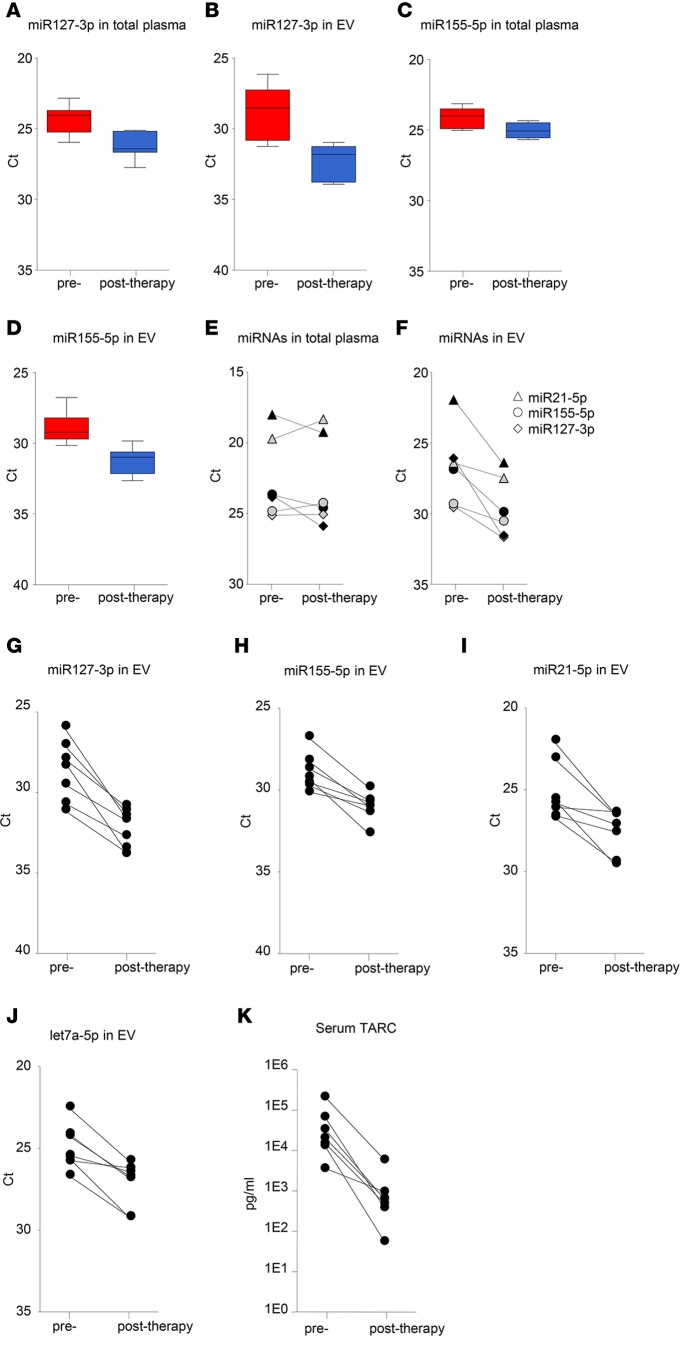
EV outperforms total plasma for monitoring treatment response and corresponds with TARC. (**A**) RT-PCR analysis of miR127-3p in total plasma of cHL patients (*n* = 7) before and after treatment, after RNA isolation using TRIzol-LS. (**B**) RT-PCR analysis of miR127-3p in plasma extracellular vesicles (EVs) of the same cHL patients (*n* = 7) as in **A**, after size-exclusion chromatography (SEC) and total RNA isolation. For each individual, the mean Ct value of SEC fractions 9 and 10 is used. Boxes show the 25%–75% percentile; whiskers show the minimum-maximum; and lines represent the median. (**C** and **D**) As in **A** and **B**, but for miR155-5p. (**E** and **F**) RT-PCR analysis of miR21-5p, miR155-5p, and miR127-3p in total plasma (**E**) and in plasma EVs (**F**) of an individual cHL patient with primary tumor before and after first-line treatment (gray symbols) and a cHL patient with relapsed disease before and after second-line treatment (black symbols). (**G**–**J**) RT-PCR analysis of miR127-3p (**G**), miR155-5p (**H**), miR21-5p (**I**), and let7a-5p (**J**) in plasma EVs of cHL patients before and after treatment (*n* = 7). Each data point is the mean Ct value of the 2 consecutive SEC fractions 9 and 10. (**K**) Serum TARC levels in the same cHL patients as in **G–J** before and after treatment, as measured by ELISA. Data are shown as paired before and after therapy samples (**E–K**).

To determine if circulating EV miRNAs outperform total plasma miRNAs in monitoring response to therapy, we analyzed samples from 7 cHL patients before and after therapy. On average miR127-3p and miR155-5p levels decreased in total plasma (4-fold and 2-fold, respectively, *P* = 0.0052 and 0.016) (Figure 8, A and C). However, the decrease was more pronounced when measured in isolated EV fractions (11-fold and 4-fold, respectively, *P* = 0.0014 and 0.0016) (Figure 8, B and D). Importantly, when examining the individual patients, plasma EV-associated miRNAs were much better suited for monitoring response to (first-line and second-line) treatment compared with total plasma. In one cHL patient with a primary tumor, miR127-3p, miR155-5p, and miR21-5p in EVs decrease markedly upon BEACOPP treatment (Figure 8F, gray), consistent with CMR. However, miR127-3p, miR155-5p, and miR21-5p levels in total plasma either remained stable or slightly increased (Figure 8E, gray). Similarly, in a relapsed cHL patient treated with second-line chemotherapy, DHAP, and brentuximab-vedotin, followed by autologous stem cell transplant, miR127-3p, miR155-5p, and miR21-5p in EVs (Figure 8F, black) decreased robustly upon treatment compared with a modest decrease of total plasma levels (Figure 8E, black). Thus, miRNAs in plasma EV fractions are suitable for response to therapy in individual cHL patients. Indeed, we observed a 3- to 10-fold reduction in miR127-3p, miR155-5p, miR21-5p, and let7a-5p levels in the EV fractions when using equal volume input (Figure 8, G–J). This corresponded with CMR, as determined by FDG-PET in 6 of 7 patients. As mentioned, in one patient who did not show CMR after 1 year, we observed an increase in miRNA levels in plasma vesicles (Figure 7, F–J).

TARC is highly expressed in HRS cells that form a small minority of the cHL tumor mass (11, 12). TARC levels are highly increased in cHL patient sera and plasma, and these levels decrease in most clinical responders (13, 14). We analyzed TARC levels using a standard ELISA assay in matched serum samples of 7 cHL patients before and after treatment with ELISA. Crucially, we observed substantial decreases in TARC levels of the same 7 patients (Figure 8K) that have decreased miR127-3p, miR155-5p, miR21-5p, and let7a-5p in plasma vesicles.

## Discussion

To improve patient care of lymphoma patients by reducing the risk of relapse, accurate knowledge of vital tumor status during treatment is necessary for informed clinical decision-making. Despite a suboptimal positive predictive value, radioactive FDG-PET is currently the method of choice for monitoring therapy response and relapse in cHL (35–37). Molecular tests that can be issued repeatedly without adverse effects might complement the imaging methods for cHL patients to improve care. We applied RNAseq and TaqMan qRT-PCR on purified plasma vesicles (EV) and protein/HDL fractions and identified a small panel of cHL-related miRNAs (miR21-5p, miR127-3p, let7a-5p, miR155-5p, and miR24-3p) in the EV fractions of patients. We tested this panel for its utility as a potential therapy response tool in primary and relapsed cHL patients. We observed that the abundance of these EV-associated miRNAs consistently and stably dropped in patients with CMR to treatment. However, in one patient receiving allogenic stem cell transplant, miRNA levels initially dropped, corresponding with FDG-PET, suggesting remission. However, miRNA levels increased after 1 year, in parallel with the reappearance of nodules in the chest (Figure 7, F–J). Our study suggests that analysis of the exRNA repertoire in EVs holds advantages over exRNA analysis from total plasma for response monitoring in cHL patients.

While ctDNA is the gold standard for liquid biopsy approaches in clinical cancer care (2, 3, 9), a potential drawback in a cHL setting is that malignant HRS cells bearing specific mutations are rare and driver mutations are infrequent (8). Moreover, the mutational landscape of cHL is diverse, and the malignant stroma is a crucial cofactor that is ignored with mutation-based ctDNA approaches (9). Apart from tumors shedding DNA fragments, intact and stabilized miRNAs are actively secreted into circulation from tumor cells (5, 25, 38). In fact, circulating plasma miRNAs were recently considered as potential therapy response biomarkers in cHL, although in this study, the candidate miRNAs were identified in cHL tissues first and then evaluated for monitoring purposes in unfractionated total plasma. The miRNA alterations measured in diseased tissues may not necessarily translate into alterations that can be measured in total plasma, in which cell-free miRNAs have a high dynamic range (39). Nondisease-specific stabilization of abundant miRNAs by proteins or heterogeneous expression lowers the sensitivity for detecting tumor-associated miRNAs (25).

We hypothesized that cHL tissues secrete a mixed population of tumor-derived EVs that bring lymphoma-associated miRNAs into circulation where they are protected from degradation. To screen for candidates, we performed RNAseq analysis on EVs from tumor cells and directly on circulating EVs that were isolated with SEC. SEC is a promising method (23–25) that yields highly enriched populations of plasma EVs (Figure 1). We “rediscovered” miR21-5p as a potential candidate miRNA that is increased in circulation and overexpressed in cHL tissues (5). We further identified miR24-3p, miR127-3p, and let7a-5p in circulating EVs that were not identified by array profiling analysis of cHL tissues (5). However, these miRNAs are expressed in multiple lymphoma cell lines as shown previously (31), including HRS cells (this study) (Figure 3A). Despite a limited sample size, we found increased concentrations of EVs in the plasma of cHL patients compared with healthy controls that were generally smaller in the size (Figure 1F). This was consistent with increased detection of EV-associated miR142-3p and let7-5p by both qRT-PCR and RNAseq (Figure 2, A and C; Figure 3, D and E; and Supplemental Figure 2D). While miR142-3p levels are not increased in EV fractions from cHL patients compared with healthy EVs (Supplemental Figure 2D), let7a-5p seems much more abundant in patient EVs (Figure 3, D and E). In fact, when we measured the candidate miRNAs, miR127-3p, miR155-5p, miR21-5p, and let7a-5p, by TaqMan RT-PCR in 3 patient samples, we observed a positive correlation of the average Ct value with the plasma EV concentration, as measured by qNano (Supplemental Figure 2C). Our RT-PCR data and particle analysis combined suggest that EVs in circulation are heterogeneous, not only in size, but also in miRNA content, which is distinct from the protein-associated miRNA repertoire (40). Moreover, when we estimate the amount of any given miRNA per vesicle we found a very similar stoichiometry as recently reported (40). We hypothesize that the concentration of lymphoma-related miRNAs decreases in the EV fractions of cHL patients during and after therapy, while nonlymphoma-related miRNAs are more stable in the EV fractions, such as miR10b-5p, mir142-3p, and miR486-5p.

The dynamic range of plasma miRNAs is a big hurdle for biomarker discovery and validation. Typically, circulating small RNAs are discovered as cancer biomarkers in miRNA arrays, as the wide range of concentrations of different miRNAs in human plasma makes detection of low abundant species with RNAseq in unfractionated plasma challenging (33, 41, 42). Our RNAseq results from small quantities of RNA input confirm that miR486-5p and miR92a-3p are highly abundant miRNAs in human plasma (Supplemental Figure 2B) (25, 41). We nevertheless identified more than 400 miRNAs in the EV fractions from cHL patients and control subjects. Overall, the miRNA diversity in plasma EVs (60–250 different miRNAs per sample) seems higher compared with the protein/HDL fractions (60–110 different miRNAs per sample) (Figure 3B), although we cannot rule out that the detection of low abundant species in protein fractions is hampered by the extreme abundance of miR486-5p and miR92a-3p. Since the miRNA diversity did not correlate with total miRNA read counts (Supplemental Figure 2E), we presume that the observed differences are not a consequence of sequencing depth alone but may be related to an underlying biology. Thus, SEC-purified EV fractions are a preferred exRNA biosource for identification of abundant and less-abundant miRNAs.

We found that EV-associated miR21-5p, miR127-3p, let7a-5p, miR24-3p, and miR155-5p signals were elevated in primary and relapsed cHL patients compared with healthy individuals. Previously, Jones and colleagues reported elevated levels of miR21 and miR155 in total plasma of cHL patients compared with healthy control plasma (5). However, these authors did not document decreased miR155 levels during or after therapy, while we detected small, but significant, decreases (2-fold, *P* = 0.016) in unfractionated plasma after therapy. Nevertheless, the decrease in miR155-5p was more pronounced in the EV fraction (4-fold, *P* = 0.016) (Figure 8, C and D). We cannot exclude that the preanalytical conditions in the Jones study (samples were processed up to 24 hours after collection) may have obscured miR155 differences in total plasma (5). Our results suggest that most candidate miRNAs for treatment response monitoring measured in EVs outperform miRNAs found in total plasma increasing sensitivity and specificity. This was certainly true for the newly identified cHL marker miR127-3p, which was not previously linked to cHL when analyzing tumor tissue. miR127-3p is functionally involved in the B cell differentiation process through posttranscriptional regulation of *BLIMP1* and *XBP1* genes (43). *BLIMP* was previously reported as a direct target of let7a in HRS cells (44) and cooperates with miR127-3p in driving lymphomagenesis (34). We detected high levels of miR127-3p in EVs produced by HRS cells in culture (Figure 3A) and in plasma EVs of cHL patients but less abundant levels in healthy control EVs (Figure 5A). ROC analysis illustrated that EV-associated miR127-3p can distinguish cHL patients from controls (AUC = 0.80), while this is not the case when using total plasma as exRNA source (AUC = 0.50) (Figure 6, C and D). This suggests that the pool of miR127-3p detected in the protein fraction is unrelated to cHL tumor tissue and is derived from other sources. It remains to be demonstrated whether miR127-3p levels in EVs reflect total expression of this miRNA in HRS cells, which are vital for the secretion of this miRNA via EVs into circulation.

This study confirms the original discovery of the value of stabilized circulating miRNAs as cancer biomarkers (25, 38) and supports the recent observation that these are suited for serial testing to assess the kinetics of response to therapy (39). The main advantage of this study is that SEC-isolated EVs from plasma are compatible with RNAseq. Using RNAseq, we found evidence that miRNA measurements in EV fractions can increase the signal-to-noise ratio of miRNA biomarkers in plasma. It is further clear that RNAseq normalization methods used for differential expression analysis in tissues need improvement, as these methods fail to identify low abundant candidate miRNAs in plasma EV and protein fractions. The procedure of isolating EVs with SEC from plasma and miRNA detection by TaqMan PCR is easy to learn and robust, and no specialized laboratory equipment is needed. Thus, our approach is, in principle, compatible with routine diagnostic testing. While not the focus here, in the future, RNAseq as a detection platform seems promising (45). Our results warrant larger studies with longitudinal patient samples to assess the clinical utility of EV miRNAs as a molecular diagnostic tool for treatment response, relapse, and potentially minimal residual disease monitoring for cHL patients.

## Methods

### Patients.

Twenty patients with cHL were included (Figure 1); patient characteristics are summarized in Table 1. Seven of twenty patients had relapsed disease at initial presentation. Interim and after treatment samples were available from 7 patients, up to 15 months after treatment initiation. Six of seven patients showed CMR, as determined by 18F FDG-PET, and 1 patient did not reach complete remission. As controls, 9 healthy donor plasma samples were used and additionally 4 patients with systemic lupus erythematosus (SLE) were included. The SLE patient group consists of patients with active disease as well as patients in remission. One patient received immune-suppressive drugs at time of analysis.

Blood was collected in K2E (EDTA) tubes and serum collection tubes (BD Vacutainer) and processed within 2 hours after collection. Platelet-free plasma and sera were isolated as described in Supplemental Figure 1A and stored in 1.5-ml aliquots at –80°C until further analysis. Freeze-thaw cycles were avoided.

### SEC.

SEC was performed as described previously (23). Sepharose CL/2B (GE Healthcare) in 0.32% citrate/PBS was stacked in a BD syringe up to a 10-ml column bed volume, and this was used to separate vesicles and protein/HDL from 1.5 ml plasma, sera, or cell culture supernatant. Immediately after applying the samples onto the column, 26 fractions of 0.5 ml were collected and stored at –80°C at least overnight until further analysis (Supplemental Figure 1B). Fractions 9 and 10 are considered as vesicle-enriched fractions, and fractions 20 and 21 as protein/HDL-enriched fractions. The fractions were processed and analyzed individually to serve as an internal duplicate. Notably, for SLE patient plasma, 0.5 ml was used for SEC, 0.32% citrate/PBS was added to adjust the volume up to 1.5 ml. Freeze-thaw cycles of the fractions were avoided. EM was done as described previously (32).

### Cell culture.

Hodgkin lymphoma cell lines (L1236, KMH2), diffuse large B cell lymphoma cell lines (Ly3, Ly10, HL4, HL5; gifts from S. Cillessen [Department of Pathology, VU University Medical Center, Amsterdam, Netherlands]), and EBV-infected lymphoblastoid cell lines (IK140508, RN) were cultured in RPMI-1640 (Lonza), supplemented with 10% FBS (Hyclone), 100 U/ml penicillin G, 100 μg/ml streptomycin sulfate, and 2 mM glutamine. Exosomes were isolated as described previously (46).

### Spike in.

B cell tumor–derived exosomes were isolated by differential ultracentrifugation as described previously (46). An EBV-infected B cell line (RN) was chosen to enable distinguishing spiked in exosomes from circulating plasma EVs. 50 μl exosomes were spiked into 1.5 ml plasma from an EBV-negative healthy donor prior to SEC. Fractions were collected and stored at –80°C until RNA analysis.

### RNA isolation.

Total RNA was isolated using TRIzol (Thermo Fisher Scientific) according to the manufacturer’s instructions, with some modifications. 0.75 ml TRIzol was added to 0.25 ml SEC fractions, mixed properly, and incubated at room temperature for 15 minutes. Samples were stored at –80°C for at least 3 hours to increase the RNA yield from vesicle fractions specifically. Prior to isopropyl precipitation 50 μg glycogen (Roche) was added. The final RNA pellet was dissolved in 10 μl nuclease-free water. For RNAseq, RNA from fractions 9 and 10 or 20 and 21 from 2.25 ml (for cHL patients) or 3.75 ml (for healthy controls) plasma was pooled in a final volume of 10 μl.

For RNA isolation of total plasma or sera, 0.75 ml TRIzol-LS (Thermo Fisher Scientific) was added to 0.25 ml plasma and further processed as described above.

### Small RNAseq and analysis.

The RNA quality from plasma EVs and protein/HDL fractions from cHL patients and healthy donors was analyzed on a Agilent 2100 Bioanalyzer using a small RNA chip. Maximum input (6 μl) was prepared for sequencing using the Illumina TruSeq small RNA Preparation Kit according to the manufacturer’s instructions. RNAseq was performed on a HiSeq 2500 (Illumina, single-end 50-bp read length), using equimolar amounts for each sample. Sequencing analysis was done by using the sRNAbench package as described previously (47). Briefly, after adapter trimming and unique read grouping, reads were aligned to the human genome (UCSC hg19) using Bowtie 1.1.2. To provide annotations for RNA elements that mapped to the human genome, several databases were used, including miRBase (version 21) for mature and pre-miRNA sequences and the NCBI Reference Sequences (RefSeq release 69, January, 2 2015). A differential expression module based on edgeR was used to generate an expression matrix of all miRNAs detected (48) using trimmed mean of M values normalization for the detection of differentially expressed small RNAs (49). RNAseq on cell line–derived exosomes was done as described previously (29).

### Multiplex stem-loop RT-PCR.

Equal volumes of RNA (3 μl) were reverse transcribed using the TaqMan MicroRNA Reverse Transcription kit (Thermo Fisher Scientific). Equal volumes were used throughout to reduce technical variation, since an optimal normalizer for small RNAs in circulation has not been established (33).

Up to 5 RT-primers (TaqMan MicroRNA Assay, Thermo Fisher Scientific) were used in a multiplex reaction after checking their sensitivity and specificity in a singleplex and multiplex reaction. After cDNA synthesis nuclease-free water was added up to a final volume of 50 μl. 3 μl of cDNA was subjected to 40 cycles of 95°C for 15 seconds and 60°C for 1 minute on a ABI 7500 Fast system. All samples were measured in duplo, and the input for PCR was 13.5 μl plasma equivalents, with the exception of SLE patient plasma (3.2–6.4 μl). Data were analyzed using 7500 Software v2.0.6, and, for comparison of miRNA levels, the Ct threshold was set at similar levels in different PCR runs. The following miRNA assays were used: miR127-3p (ID 000452), miR155-5p (ID 002623), miR21-5p (ID 000397), let7a-5p (ID 000377), miR24-3p (ID 000402), miR10b-5p (ID 002218), miR142-3p (ID 000464), miR92a-3p (ID 000431), and miR451-5p (ID 001105) (Thermo Fisher Scientific). ncRNA vtRNA1-1 and EBV miRNAs BHRF1-3 and BART2-5p were amplified as described previously (32, 50).

### TARC analysis.

Serum TARC was measured using a double antibody sandwich ELISA (R&D Systems) as described previously (13).

### High-resolution flow cytometry.

High-resolution flow cytometry was performed as described previously (28). Briefly, SEC fractions were fluorescently labeled with the lipid dye PKH67 (Sigma-Aldrich), and EVs were floated to their buoyant density by sucrose gradient flotation. Gradient fractions were subsequently analyzed for EV content by performing fluorescent threshold triggering on a BD Influx flow cytometer that was adapted for analysis of nano-sized particles.

### Particle analysis by TRPS.

The concentration and diameter of circulating vesicles were determined by TRPS using a qNano device (iZON). Samples were analyzed using 150-nm pore size and the average result of pressures of 3, 5, and 8 mbar. All samples were measured in duplicate.

### Statistics.

A Kolmogorv-Smirnov test was used to check the Gaussian distribution, followed by unpaired 2-tailed *t* test. Analyses were performed using Graphpad Prism 6 software. *P* values of less than 0.05 were considered significant. Leave-one-out cross-validation was used to produce predictions of disease status per individual using a logistic regression model fitted to all remaining individuals, except the one predicted. Using the individual predicted probabilities, ROC curves and corresponding AUCs were produced per miRNA.

### Study approval.

Clinical investigation was conducted according to the Declaration of Helsinki and approved by the VU University Medical Center medical ethical committee. All samples were obtained with written informed consent.

## Author contributions

MAJVE, DDJ, JMZ, and DMP designed the study with the help of RN and AVDB. MAJVE performed the experiments with assistance from NJG, SVN, SRB, and DKL. HVDV designed the qNano studies. MHMW and SFWML performed high-resolution FACS. LV performed TARC assays. JRTVW performed EM analysis. RDXM and MAJVE performed statistical analysis. MAJVE, EEED, and DMP analyzed data and wrote the manuscript with contributions from all authors.

## Supplementary Material

Supplemental data

ICMJE disclosure forms

## Figures and Tables

**Table 1:**
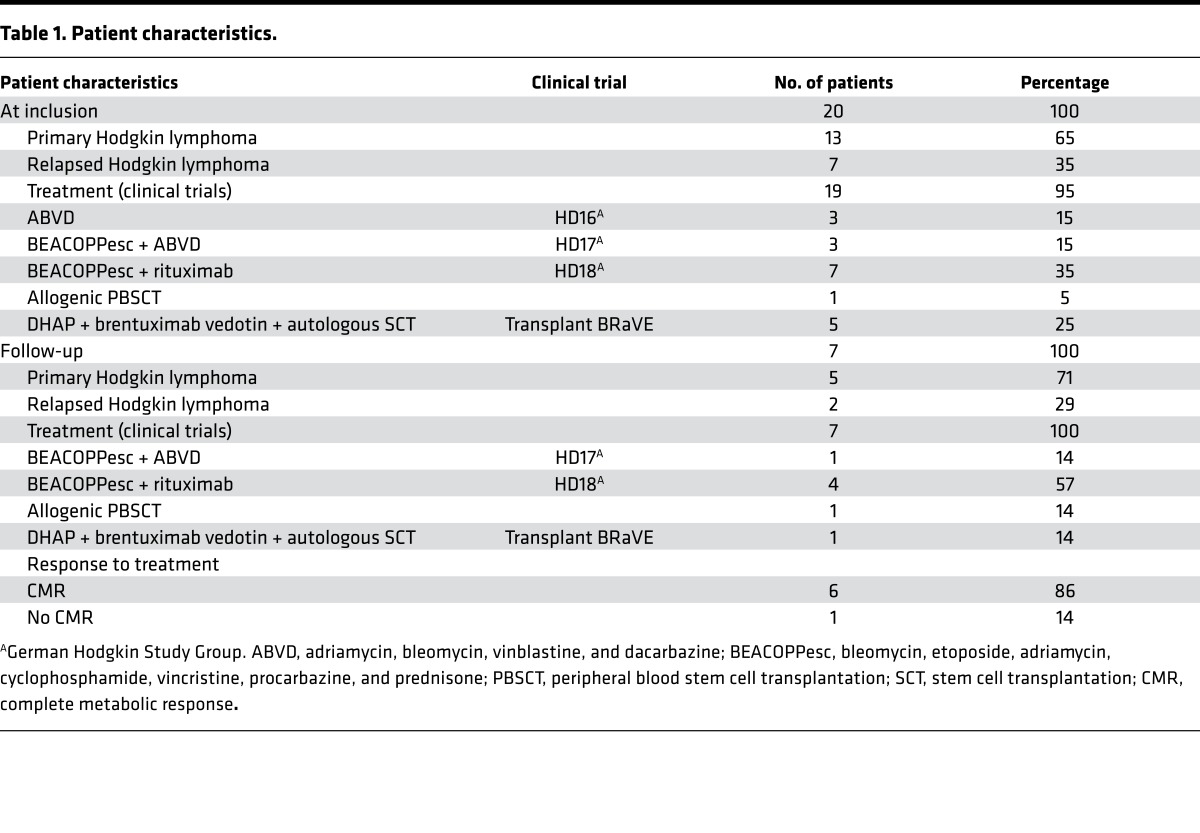
Patient characteristics.

## References

[1] Cortez MA, Bueso-Ramos C, Ferdin J, Lopez-Berestein G, Sood AK, Calin GA (2011). MicroRNAs in body fluids--the mix of hormones and biomarkers. Nat Rev Clin Oncol.

[2] Bettegowda C (2014). Detection of circulating tumor DNA in early- and late-stage human malignancies. Sci Transl Med.

[3] Diehl F (2008). Circulating mutant DNA to assess tumor dynamics. Nat Med.

[4] Kurtz DM (2015). Noninvasive monitoring of diffuse large B-cell lymphoma by immunoglobulin high-throughput sequencing. Blood.

[5] Jones K, Nourse JP, Keane C, Bhatnagar A, Gandhi MK (2014). Plasma microRNA are disease response biomarkers in classical Hodgkin lymphoma. Clin Cancer Res.

[6] Sozzi G (2014). Clinical utility of a plasma-based miRNA signature classifier within computed tomography lung cancer screening: a correlative MILD trial study. J Clin Oncol.

[7] Best MG (2015). RNA-Seq of tumor-educated platelets enables blood-based pan-cancer, multiclass, and molecular pathway cancer diagnostics. Cancer Cell.

[8] Küppers R, Engert A, Hansmann ML (2012). Hodgkin lymphoma. J Clin Invest.

[9] Schwarzenbach H, Hoon DS, Pantel K (2011). Cell-free nucleic acids as biomarkers in cancer patients. Nat Rev Cancer.

[10] Cheson BD (2011). Role of functional imaging in the management of lymphoma. J Clin Oncol.

[11] van den Berg A, Visser L, Poppema S (1999). High expression of the CC chemokine TARC in Reed-Sternberg cells. A possible explanation for the characteristic T-cell infiltratein Hodgkin’s lymphoma. Am J Pathol.

[12] Cuccaro A (2016). CD68+ cell count, early evaluation with PET and plasma TARC levels predict response in Hodgkin lymphoma. Cancer Med.

[13] Plattel WJ (2012). Plasma thymus and activation-regulated chemokine as an early response marker in classical Hodgkin’s lymphoma. Haematologica.

[14] Jones K (2013). Serum CD163 and TARC as disease response biomarkers in classical Hodgkin lymphoma. Clin Cancer Res.

[15] Witwer KW (2015). Circulating microRNA biomarker studies: pitfalls and potential solutions. Clin Chem.

[16] Kluiver J (2005). BIC and miR-155 are highly expressed in Hodgkin, primary mediastinal and diffuse large B cell lymphomas. J Pathol.

[17] Nakagawa R (2016). MicroRNA-155 controls affinity-based selection by protecting c-MYC+ B cells from apoptosis. J Clin Invest.

[18] Valadi H, Ekström K, Bossios A, Sjöstrand M, Lee JJ, Lötvall JO (2007). Exosome-mediated transfer of mRNAs and microRNAs is a novel mechanism of genetic exchange between cells. Nat Cell Biol.

[19] Skog J (2008). Glioblastoma microvesicles transport RNA and proteins that promote tumour growth and provide diagnostic biomarkers. Nat Cell Biol.

[20] Zomer A, Vendrig T, Hopmans ES, van Eijndhoven M, Middeldorp JM, Pegtel DM (2010). Exosomes: Fit to deliver small RNA. Commun Integr Biol.

[21] Zhang L (2015). Microenvironment-induced PTEN loss by exosomal microRNA primes brain metastasis outgrowth. Nature.

[22] Köberle V (2013). Differential stability of cell-free circulating microRNAs: implications for their utilization as biomarkers. PLoS One.

[23] Böing AN, van der Pol E, Grootemaat AE, Coumans FA, Sturk A, Nieuwland R (2014). Single-step isolation of extracellular vesicles by size-exclusion chromatography. J Extracell Vesicles.

[24] Welton JL, Webber JP, Botos LA, Jones M, Clayton A (2015). Ready-made chromatography columns for extracellular vesicle isolation from plasma. J Extracell Vesicles.

[25] Arroyo JD (2011). Argonaute2 complexes carry a population of circulating microRNAs independent of vesicles in human plasma. Proc Natl Acad Sci U S A.

[26] Lobb RJ (2015). Optimized exosome isolation protocol for cell culture supernatant and human plasma. J Extracell Vesicles.

[27] Lozano-Ramos I (2015). Size-exclusion chromatography-based enrichment of extracellular vesicles from urine samples. J Extracell Vesicles.

[28] van der Vlist EJ, Nolte-’t Hoen EN, Stoorvogel W, Arkesteijn GJ, Wauben MH (2012). Fluorescent labeling of nano-sized vesicles released by cells and subsequent quantitative and qualitative analysis by high-resolution flow cytometry. Nat Protoc.

[29] Koppers-Lalic D (2014). Nontemplated nucleotide additions distinguish the small RNA composition in cells from exosomes. Cell Rep.

[30] Gibcus JH (2009). Hodgkin lymphoma cell lines are characterized by a specific miRNA expression profile. Neoplasia.

[31] Jima DD (2010). Deep sequencing of the small RNA transcriptome of normal and malignant human B cells identifies hundreds of novel microRNAs. Blood.

[32] Pegtel DM (2010). Functional delivery of viral miRNAs via exosomes. Proc Natl Acad Sci U S A.

[33] Freedman JE (2016). Diverse human extracellular RNAs are widely detected in human plasma. Nat Commun.

[34] Robertus JL (2009). Specific expression of miR-17-5p and miR-127 in testicular and central nervous system diffuse large B-cell lymphoma. Mod Pathol.

[35] von Tresckow B (2012). Dose-intensification in early unfavorable Hodgkin’s lymphoma: final analysis of the German Hodgkin Study Group HD14 trial. J Clin Oncol.

[36] Zijlstra JM, Boellaard R, Hoekstra OS (2009). Interim positron emission tomography scan in multi-center studies: optimization of visual and quantitative assessments. Leuk Lymphoma.

[37] Cheson BD (2014). Recommendations for initial evaluation, staging, and response assessment of Hodgkin and non-Hodgkin lymphoma: the Lugano classification. J Clin Oncol.

[38] Mitchell PS (2008). Circulating microRNAs as stable blood-based markers for cancer detection. Proc Natl Acad Sci U S A.

[39] Ward J (2014). Circulating microRNA profiles in human patients with acetaminophen hepatotoxicity or ischemic hepatitis. Proc Natl Acad Sci U S A.

[40] Chevillet JR (2014). Quantitative and stoichiometric analysis of the microRNA content of exosomes. Proc Natl Acad Sci U S A.

[41] Williams Z (2013). Comprehensive profiling of circulating microRNA via small RNA sequencing of cDNA libraries reveals biomarker potential and limitations. Proc Natl Acad Sci U S A.

[42] Sterling CH, Veksler-Lublinsky I, Ambros V (2015). An efficient and sensitive method for preparing cDNA libraries from scarce biological samples. Nucleic Acids Res.

[43] Leucci E (2010). B-cell differentiation in EBV-positive Burkitt lymphoma is impaired at posttranscriptional level by miRNA-altered expression. Int J Cancer.

[44] Nie K (2008). MicroRNA-mediated down-regulation of PRDM1/Blimp-1 in Hodgkin/Reed-Sternberg cells: a potential pathogenetic lesion in Hodgkin lymphomas. Am J Pathol.

[45] Byron SA, Van Keuren-Jensen KR, Engelthaler DM, Carpten JD, Craig DW (2016). Translating RNA sequencing into clinical diagnostics: opportunities and challenges. Nat Rev Genet.

[46] Verweij FJ, van Eijndhoven MA, Middeldorp J, Pegtel DM (2013). Analysis of viral microRNA exchange via exosomes in vitro and in vivo. Methods Mol Biol.

[47] (2014). sRNAbench: profiling of small RNAs and its sequence variants in single or multi-species high-throughput experiments. Methods Next Gener Seq.

[48] Robinson MD, McCarthy DJ, Smyth GK (2010). edgeR: a Bioconductor package for differential expression analysis of digital gene expression data. Bioinformatics.

[49] Robinson MD, Oshlack A (2010). A scaling normalization method for differential expression analysis of RNA-seq data. Genome Biol.

[50] Baglio SR (2016). Sensing of latent EBV infection through exosomal transfer of 5’pppRNA. Proc Natl Acad Sci U S A.

