# Supplementary material for: Plasma vesicle miRNAs for therapy response monitoring in Hodgkin lymphoma patients
Source: JCI Insight. 2016 Nov 17;1(19):e89631. doi: 10.1172/jci.insight.89631 (PMC5111516; doi:10.1172/jci.insight.89631)
Supplement: Supplemental data [file jciinsight-1-89631-s001.pdf]

Suppl. Figure 1

Plasma is preferred over serum for small RNA analysis using SEC

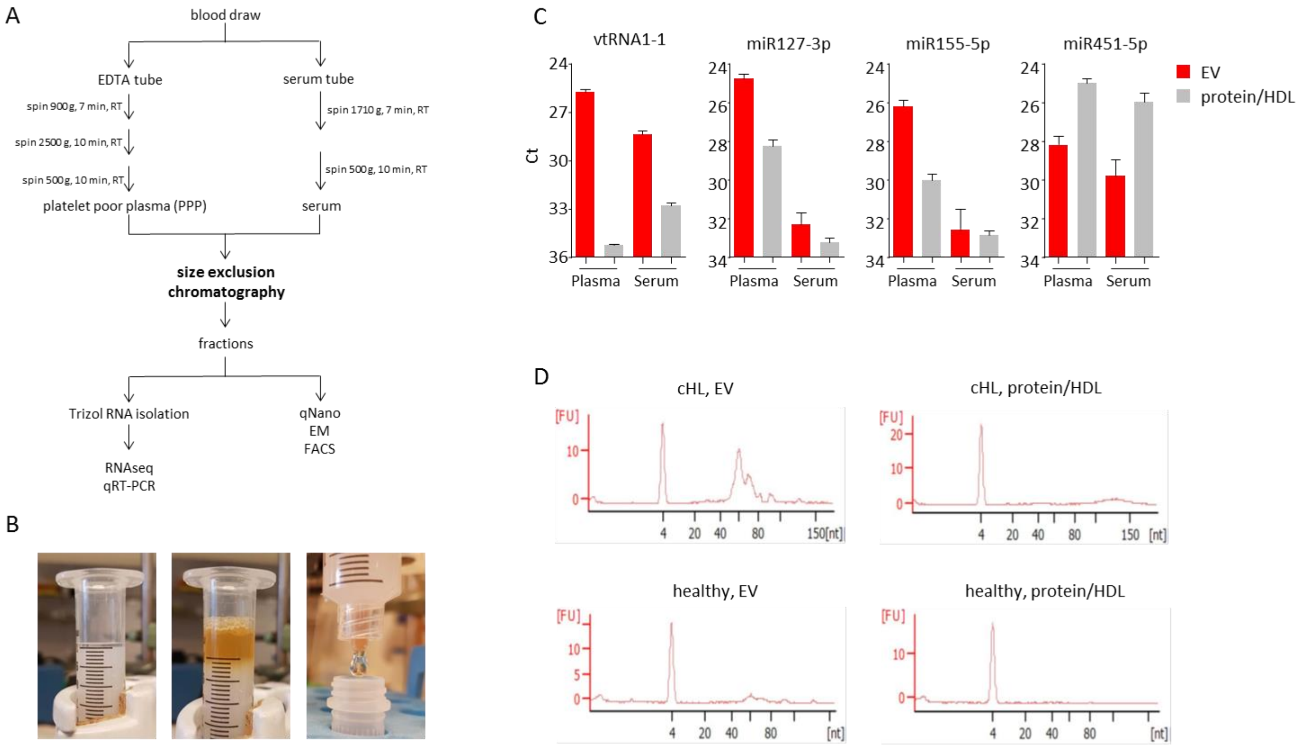

A) Schematic overview of methods used for plasma and serum isolation and subsequent experiments. B) Images of size-exclusion chromatography (SEC) before (left) and after (middle) applying plasma and flow-through (right). C) vtRNA1-1, miR127-3p, miR155-5p and miR451-5p in cHL patient plasma or serum after SEC. Data shown are from one representative donor (n=2) and depicted as mean Ct values +/- SEM of the 2 consecutive SEC fractions. D) Small RNA Bioanalyzer profiles of cHL (upper panel) and healthy donor (lower panel) plasma extracellular vesicles EV (left) and protein/HDL fraction (right). Profiles shown are of one representative donor (n=3).

Suppl. Figure 2

RNAseq and particle analysis in plasma EVs and protein/HDL fractions

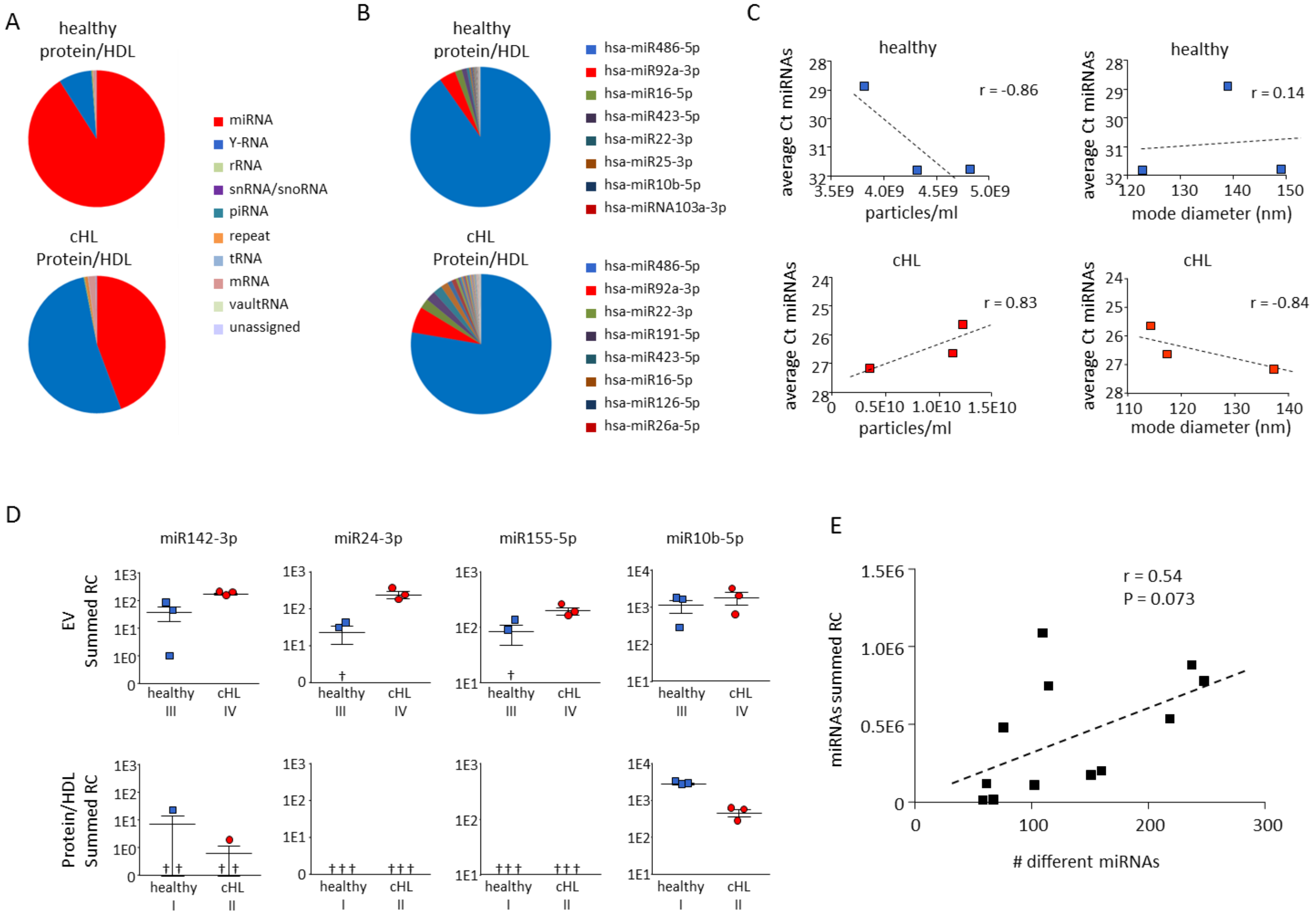

A) Distribution of small RNA sub classes in plasma protein/HDL fractions of a healthy individual (upper panel) and a cHL patient (lower panel). Data shown are of one representative donor (n=3) and depicted as % read counts. B) Distribution of individual miRNA species in protein/HDL fractions of a healthy donor (upper panel) and a cHL patient (lower panel). Data shown are of one representative donor (n=3) and depicted as % of miRNA read counts. C) Correlation between miRNA level and qNano particle concentration (left) and mode diameter (right) of healthy plasma extracellular vesicles (EVs) (upper panel) and cHL patient EVs (lower panel) (n=3 each). MiRNA level is based on the average Ct value (qRT-PCR) of miR127-3p, miR155-5p, miR21-5p and let7a-5p. r=Pearson correlation. D) RNAseq analysis of

miR142-3p, miR24-3p, miR155-5p and miR10b-5p in healthy and cHL patient plasma EVs (upper panel) and protein/HDL fractions (lower panel). Dots represent individual samples and lines are mean +/- SEM. Data are shown as summed read counts. †=not detected. E) Correlation between miRNA summed read counts and the number of different miRNAs detected by RNAseq in the plasma EV and protein/HDL fractions of healthy donors and cHL patients (n=12 samples). r=Pearson correlation. P value is calculated using an unpaired two tailed t test.

Suppl. Figure 3

Candidate miRNA markers are disease specific

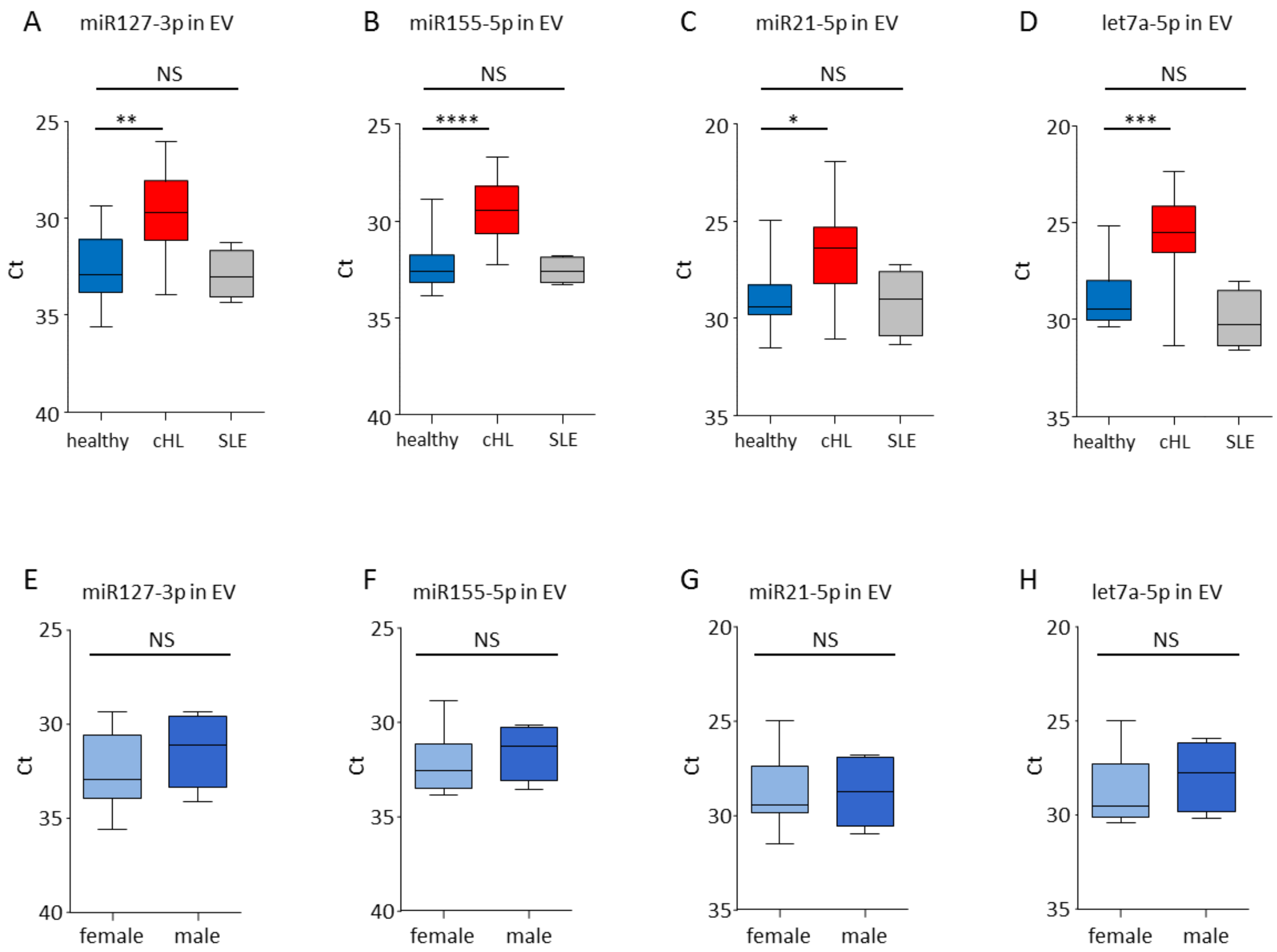

A-D) RT-PCR analysis of miR127-3p (A), miR155-5p (B), miR21-5p (C) and let7a-5p (D) in plasma extracellular vesicles (EVs) of healthy individuals (n=9), cHL patients (n=20) and SLE patients (n=4) after size-exclusion chromatography (SEC) and total RNA isolation using Trizol®. E-H) RT-PCR analysis of miR127-3p (E), miR155-5p (F), miR21-5p (G) and let7a-5p (H) in plasma EVs of male (n=6) and female (n=11) healthy individuals. For each individual the mean Ct value of SEC fractions 9 and 10 is used. Boxes show 25-75% percentile, whiskers show minimum-maximum and line represents the median. NS=not significant; * $P<0.05$; ** $P<0.005$; *** $P<0.0005$; **** $P<0.0001$ (unpaired two tailed t test).

Suppl. Figure 4

Candidate miRNA levels in EVs from relapsed cHL and levels normalized to miR10b-5p

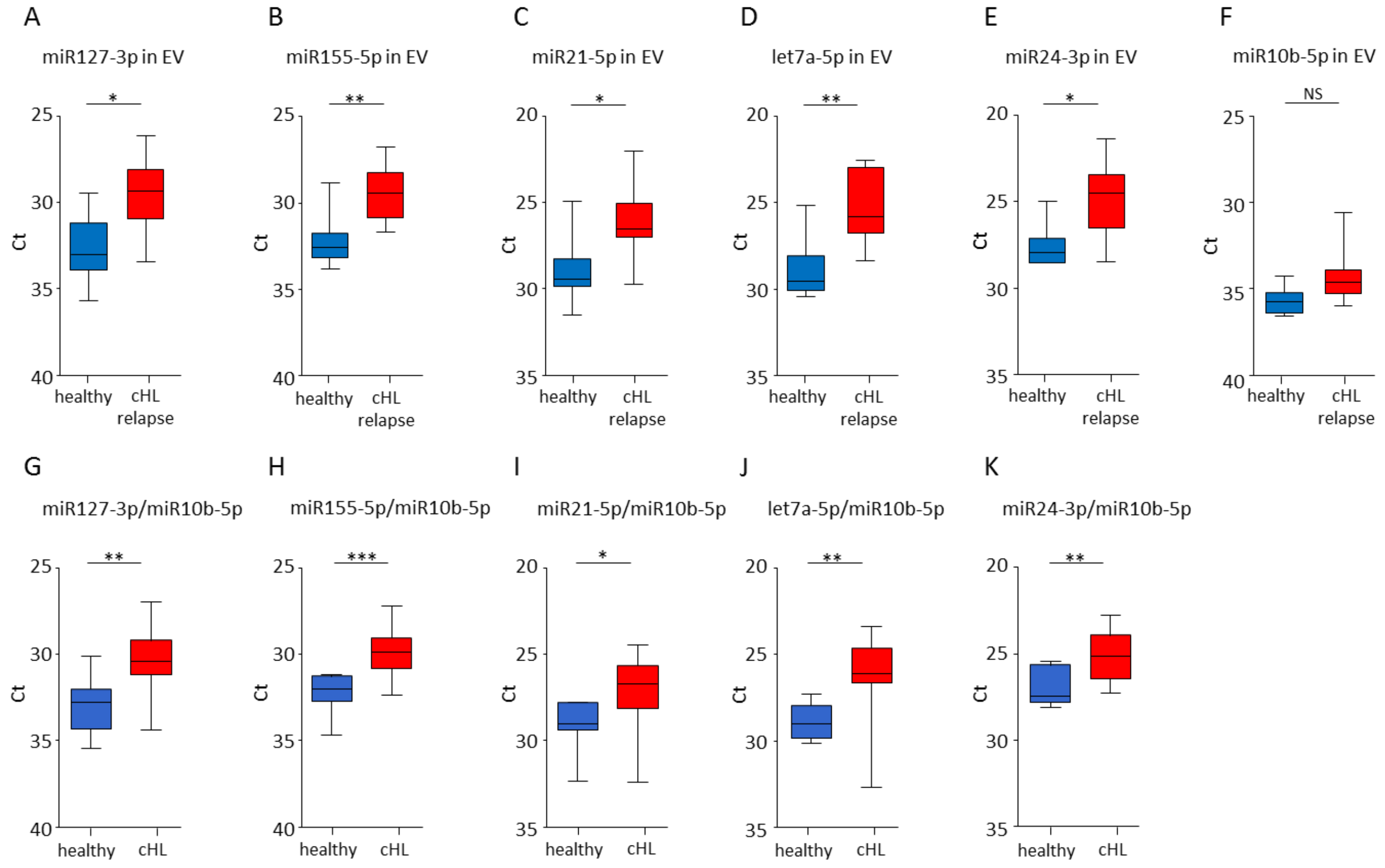

A-F) RT-PCR analysis of miR127-3p (A), miR155-5p (B), miR21-5p (C), let7a-5p (D), miR24-3p (E) and miR10b-5p (F) in plasma extracellular vesicles (EVs) of healthy individuals (n=9) and cHL patients with relapsed disease (n=7) after size-exclusion chromatography (SEC) and total RNA isolation using TRIzol®. G-K) RT-PCR analysis of miR127-3p (G), miR155-5p (H), miR21-5p (I), let7a-5p (J) and miR24-3p (K) in plasma EV of healthy individuals (n=7) and cHL patients (n=20), normalized to miR10b-5p. For each individual the mean Ct value of SEC fractions 9 and 10 is used. Boxes show 25-75% percentile, whiskers show minimum-maximum and line represents the median. NS=not significant; * P<0.05; ** P<0.005; *** P<0.0005 (unpaired two tailed t test).

Suppl. Figure 5

MiR155-5p in EV outperforms total plasma and serum for distinguishing cHL patients from controls

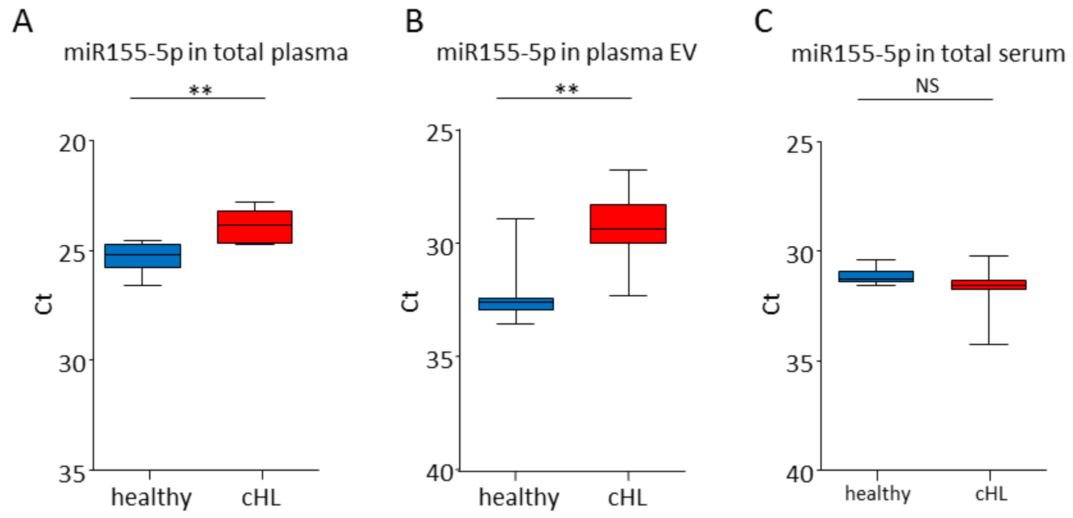

A) RT-PCR analysis of miR155-5p in total plasma of healthy controls (n=7) and cHL patients (n=8) after RNA isolation using TRIzol-LS®. B) RT-PCR analysis of miR155-5p in extracellular vesicle (EV) fractions of the same healthy individuals and cHL patients as in (A) after size-exclusion chromatography (SEC) and total RNA isolation. For each individual the mean Ct value of SEC fractions 9 and 10 is used. C) RT=PCR analysis of miR155-5p in total serum of healthy individuals (n=8) and cHL patients (n=16). Boxes show 25-75% percentile, whiskers show minimum-maximum and line represents the median. NS=not significant; ** P<0.005 (unpaired two tailed t test).

Suppl Table 1

RNAseq data

group	sample	total RC	miRNA RC	# different miRNAs	top 10 miRNA	RC	%-age	group	sample	total RC	miRNA RC	# different miRNAs	top 10 miRNA	RC	%-age
**I**	1	1318571	1092693	109	hsa-miR-486-5p	980399	89,72	**III**	1	521501	183334	150	hsa-miR-486-5p	106986	58,36
**healthy donors**					hsa-miR-92a-3p	41493	3,80	**healthy donors**					hsa-miR-92a-3p	18981	10,35
**protein/HDL**					hsa-miR-16-5p	17527	1,60	**EV**					hsa-miR-16-5p	5673	3,09
					hsa-miR-423-5p	12479	1,14						hsa-miR-26a-5p	4887	2,67
					hsa-miR-22-3p	6252	0,57						hsa-miR-22-3p	4274	2,33
					hsa-miR-25-3p	4052	0,37						hsa-miR-423-5p	3397	1,85
					hsa-miR-10b-5p	3964	0,36						hsa-miR-126-5p	2991	1,63
					hsa-miR-103a-3p	2086	0,19						hsa-let-7f-5p	2925	1,60
					hsa-miR-126-5p	1725	0,16						hsa-miR-10b-5p	2106	1,15
					hsa-miR-10a-5p	1447	0,13						hsa-miR-191-5p	1907	1,04
**I**	2	594752	490501	75	hsa-miR-486-5p	465055	94,81	**III**	2	1018257	206465	159	hsa-miR-486-5p	102868	49,82
**healthy donors**					hsa-miR-92a-3p	4760	0,97	**healthy donors**					hsa-miR-92a-3p	28137	13,63
**protein/HDL**					hsa-miR-423-5p	3274	0,67	**EV**					hsa-let-7a-5p	7954	3,85
					hsa-miR-191-5p	3250	0,66						hsa-miR-26a-5p	6737	3,26
					hsa-miR-10b-5p	3198	0,65						hsa-let-7f-5p	5611	2,72
					hsa-miR-16-5p	1924	0,39						hsa-miR-423-5p	4936	2,39
					hsa-miR-22-3p	1358	0,28						hsa-miR-191-5p	4590	2,22
					hsa-miR-10a-5p	869	0,18						hsa-miR-126-5p	4458	2,16
					hsa-miR-25-3p	848	0,17						hsa-miR-22-3p	3968	1,92
					hsa-miR-181a-5p	408	0,08						hsa-miR-181a-5p	2776	1,34
**I**	3	947357	752704	114	hsa-miR-486-5p	679700	90,30	**III**	3	73266	23251	58	hsa-miR-486-5p	16226	69,79
**healthy donors**					hsa-miR-92a-3p	17673	2,35	**healthy donors**					hsa-miR-92a-3p	1162	5,00
**protein/HDL**					hsa-miR-16-5p	11586	1,54	**EV**					hsa-miR-191-5p	914	3,93
					hsa-miR-423-5p	5939	0,79						hsa-let-7a-5p	895	3,85
					hsa-miR-22-3p	4937	0,66						hsa-miR-26a-5p	366	1,57
					hsa-miR-191-5p	3498	0,46						hsa-miR-10b-5p	333	1,43
					hsa-miR-10b-5p	3387	0,45						hsa-miR-423-5p	304	1,31
					hsa-miR-25-3p	2386	0,32						hsa-miR-126-5p	304	1,31
					hsa-miR-103a-3p	1436	0,19						hsa-let-7f-5p	288	1,24
					hsa-miR-10a-5p	1416	0,19						hsa-miR-22-3p	241	1,04
**II**	1	312559	120404	102	hsa-miR-486-5p	91499	75,99	**IV**	1	2611443	544336	217	hsa-miR-486-5p	140709	25,85
**cHL patients**					hsa-miR-92a-3p	7102	5,90	**cHL patients**					hsa-let-7a-5p	74359	13,66
**protein/HDL**					hsa-miR-22-3p	2682	2,23	**EV**					hsa-miR-92a-3p	64990	11,94
					hsa-miR-191-5p	2645	2,20						hsa-let-7f-5p	37157	6,83
					hsa-miR-423-5p	2477	2,06						hsa-miR-191-5p	27219	5,00
					hsa-miR-16-5p	1964	1,63						hsa-miR-26a-5p	21708	3,99
					hsa-miR-126-5p	1216	1,01						hsa-miR-423-5p	17143	3,15
					hsa-miR-26a-5p	1206	1,00						hsa-miR-126-5p	15374	2,82
					hsa-miR-10b-5p	655	0,54						hsa-miR-22-3p	15078	2,77
					hsa-miR-146a-5p	460	0,38						hsa-let-7b-5p	14081	2,59
**II**	2	83318	26493	67	hsa-miR-486-5p	17030	64,28	**IV**	2	2350581	886790	236	hsa-miR-486-5p	279141	31,48
**cHL patients**					hsa-miR-191-5p	3066	11,57	**cHL patients**					hsa-let-7a-5p	162100	18,28
**protein/HDL**					hsa-miR-92a-3p	951	3,59	**EV**					hsa-miR-191-5p	78956	8,90
					hsa-miR-22-3p	720	2,72						hsa-miR-92a-3p	57971	6,54
					hsa-miR-26a-5p	492	1,86						hsa-let-7f-5p	42022	4,74
					hsa-miR-423-5p	331	1,25						hsa-miR-26a-5p	25769	2,91
					hsa-miR-10b-5p	319	1,20						hsa-miR-22-3p	21390	2,41
					hsa-miR-126-5p	309	1,17						hsa-miR-126-5p	20596	2,32
					hsa-let-7a-5p	262	0,99						hsa-let-7b-5p	14365	1,62
					hsa-miR-10a-5p	242	0,91						hsa-miR-181a-5p	13030	1,47
**II**	3	190459	126160	61	hsa-miR-486-5p	115361	91,44	**IV**	3	5452409	790800	246	hsa-miR-486-5p	312111	39,47
**cHL patients**					hsa-miR-92a-3p	2906	2,30	**cHL patients**					hsa-miR-92a-3p	100836	12,75
**protein/HDL**					hsa-miR-423-5p	1571	1,25	**EV**					hsa-let-7a-5p	61007	7,71
					hsa-miR-191-5p	1091	0,86						hsa-miR-26a-5p	33116	4,19
					hsa-miR-16-5p	904	0,72						hsa-let-7f-5p	31413	3,97
					hsa-miR-10b-5p	713	0,57						hsa-miR-191-5p	28523	3,61
					hsa-miR-22-3p	590	0,47						hsa-miR-423-5p	25431	3,22
					hsa-miR-25-3p	254	0,20						hsa-let-7b-5p	18442	2,33
					hsa-miR-126-5p	232	0,18						hsa-miR-22-3p	17135	2,17
					hsa-miR-26a-5p	194	0,15						hsa-miR-126-5p	14939	1,89
